# Regulation of paternal 5mC oxidation and H3K9me2 asymmetry by ERK1/2 in mouse zygotes

**DOI:** 10.1186/s13578-022-00758-x

**Published:** 2022-03-07

**Authors:** Baobao Chen, Mingtian Deng, Meng-Hao Pan, Shao-Chen Sun, Honglin Liu

**Affiliations:** College of Animal Science and Technology, Nanjing Agricultural University, Nanjing, 210095 China

**Keywords:** ERK1/2, H3K9me2, 5mC, Paternal pronucleus, Zygotic reprogramming

## Abstract

**Background:**

Extracellular-signal-regulated kinase (ERK) direct cell fate determination during the early development. The intricate interaction between the deposition of H3K9me2, de novo 5mC, and its oxides affects the remodeling of zygotic epigenetic modification. However, the role of fertilization-dependent ERK in the first cell cycle during zygotic reprogramming remains elusive.

**Methods:**

In the present study, we used the small molecule inhibitor to construct the rapid ERK1/2 inactivation system in early zygotes in mice. The pronuclear H3K9me2 deposition assay and the pre-implantation embryonic development ability were assessed to investigate the effect of fertilization-dependent ERK1/2 on zygotic reprogramming and developmental potential. Immunofluorescence and RT-PCR were performed to measure the 5mC or its oxides and H3K9me2 deposition, and the expression of related genes.

**Results:**

We reported that zygotic ERK1/2 inhibition impaired the development competence of pre-implantation embryos. Following the ERK1/2 inhibition, H3K9me2, as well as 5mC and its oxides, were all accumulated abnormally, and the excess accumulation of paternal H3K9me2 and 5mC resulted in reduced asymmetry between parental pronuclei. Furthermore, ERK1/2 inhibition triggered paternal pronuclear localization of the H3K9 methyltransferase G9a and Tet methylcytosine dioxygenase 3 (Tet3). Moreover, the excess localization of G9a antagonized the tight binding of Tet3 to paternal chromatin when ERK1/2 was inhibited.

**Conclusions:**

In conclusion, we propose that zygotic H3K9me2 and 5mC are regulated by fertilization-dependent ERK1/2, which contributes to the development competence of pre-implantation embryos in mice.

**Supplementary Information:**

The online version contains supplementary material available at 10.1186/s13578-022-00758-x.

## Background

The paternal and maternal pronuclei show asymmetric epigenetic marks [1], chromatin structure [2, 3], and transcriptional activity [4, 5] in the zygotic reprogramming. For example, the paternal pronucleus exhibits higher transcriptional activity [4, 5], and less condensed chromatin than the maternal pronucleus in mice [2, 6]. During the zygotic reprogramming, the paternal genome initiates widespread deposition of de novo epigenetic marks, making it an excellent model to understand how epigenetic marks are loaded on the genome orderly [1, 2, 7].

In mammals, mitogen-activated protein kinases (MAPKs) regulate the stability of the maternal and zygotic transcripts [8] and are essential for the early development of mouse zygotes [9]. It was reported that the MII-phase oocytes are stabilized by appropriate levels of MPF [10] and MAPKs [11]. After sperm-oocyte fusion, the decrease of MPF activity (within 10 min) precedes that of MAPKs activities [12–14]. Recently, ERK signal was reported to direct fate specification of embryonic stem cells and the inhibition of ERK promotes enhanced stabilization of Nanog protein after mitosis [15]. However, the mechanisms by which zygotic ERK controls gene expression to influence developmental pattern formation and the quantitative understanding of these mechanisms remain unknown [16].

H3K9me2 is associated with the compact chromatin and the transcriptionally repressive state [17, 18]. It was reported that the zygotic genome exhibits an asymmetric H3K9me2 pattern. The maternal pronuclei remain considerable H3K9me2, while paternal pronuclei bear no H3K9me2 [19–21]. Subsequently, the paternal genome progressively gains H3K9me2 from the late zygote to the cleavage stage [19, 21], suggesting fine regulation of H3K9me2 during zygotic reprogramming. G9a (also known as EHMT2 and KMT1C) is responsible for this progressive deposition of paternal H3K9me2 [21], and it protects the maternal pronucleus from 5mC oxidation [22]. During embryogenesis, G9a controls transcription levels of zygotic genes [23]. These studies suggest that G9a plays pivotal roles during zygotic reprogramming [21, 22].

5mC is one of the well-documented epigenetic factors associated with gene silencing and plays an important role in facilitating the propagation of cellular identity through cell divisions [24, 25]. Meanwhile, 5-hydroxymethylcytosine (5hmC), the oxide of 5mC [26], is positively correlated with gene expression and plays an important role in epigenetic regulation and genome reprogramming during mammalian development [1, 7, 25, 27]. It was reported that the sperm-derived genome undergoes actively demethylated through Tet3-mediated 5mC oxidation, whereas the maternal genome is passive dilution by DNA replication [28, 29]. Importantly, Tet3 also contributes to paternal demethylation by counteracting the de novo 5mC [30]. While immunostaining results support a role for H3K9me2 in protecting 5mC [31, 32], many bisulfite sequencing (BS-seq) results show that 5mC in mammals is largely independent of H3K9me2 [33, 34], suggesting unknown factors regulated the cross-talk between H3K9me2 and 5mC.

Therefore, in the present study, we examined the role of zygotic ERK1/2 during preimplantation development and reported the fine regulation of paternal 5mC oxidation and H3K9me2 asymmetry by ERK1/2, which provide insights into the role of ERK1/2 during zygotic reprogramming.

## Results

### Zygotic ERK1/2 is essential for preimplantation development

**Fig. 1:**
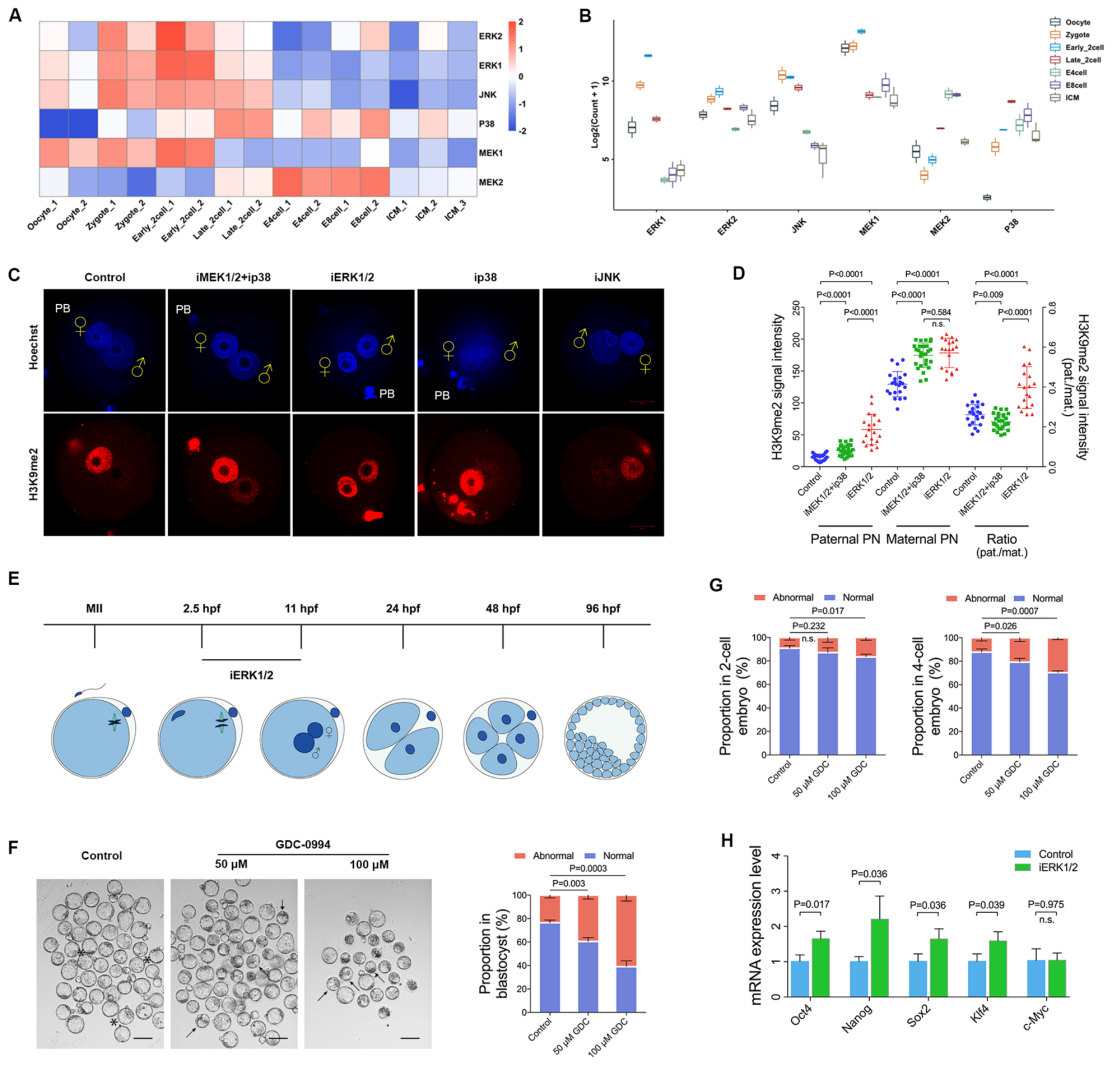
The zygotic ERK1/2 is essential for preimplantation development. **A**, **B** Heatmap and boxplot revealed dynamic expression of MAPKs during early embryo development. **C** H3K9me2 deposition in zygotes treated with inhibitors of different MAPKs pathways at PN4–5 (10 hpf). DNA is stained using Hoechst33342 (blue). Scale bars, 20 µm. **D** Quantification is represented as the mean of H3K9me2 signal intensity in pronuclei after background subtraction (left axis) or a ratio between parental pronuclei signals (pat./mat., right axis). Each data point represents an independent zygote. Number of zygotes analysed for each group: control n = 22; iMEK1/2 + ip38 n = 27; iERK1/2 (100 μM GDC-0994) n = 19. **E** Time scheme of zygote collection and embryo recovery. **F** Representative bright-field images of blastocysts recovered from the control (left), the 50 μM (middle), and 100 μM (right) GDC-0994-treated group. Black asterisks indicate examples with normal morphology; black arrows denote the abnormal embryos with the reduced cavity. Scale bars, 100 µm. **G** Percentage of abnormal embryos after ERK1/2 inhibition. From three independent experiments (total number of embryos analysed: 2-cell embryos, n = 205 for Control, n = 193 for 50 μM GDC-0994 treatment, n = 209 for 100 μM GDC-0994 treatment; 4-cell embryos, n = 198 for Control, n = 176 for 50 μM GDC-0994 treatment, n = 176 for 100 μM GDC-0994 treatment; blastocysts, n = 172 for Control, n = 134 for 50 μM GDC-0994 treatment, n = 97 for 100 μM GDC-0994 treatment). **H** Upregulation of *Oct4*, *Nanog*, *Sox2*, and *Klf4* in GDC-0994 treatment blastocysts as revealed by quantitative PCR. P values are indicated. Error bars indicate SD. ♀, maternal pronucleus; ♂, paternal pronucleus. PB, polar body

We first analyzed the expression of MAPKs during early embryo development by re-analysis the RNA-seq datasets of mouse embryos. As shown in Fig. 1A, B, MAPKs, specifically ERK1/2, were highly expressed during zygotic genome activation (ZGA), suggesting that MAPKs might play pivotal roles during ZGA in mice. To confirm the hypothesis, U0126, GDC-0994, SB203580, and SP600125 were used to inhibit the protein of MEK1/2, ERK1/2, p38, and JNK, respectively. We selected the maximum concentration before the morphology of zygotes becomes abnormal as the working concentration for short-term treatment (Additional file 1: Fig. S1), and the suppression effect of these inhibitors at the working concentration has been verified (Additional file 1: Fig. S2). As H3K9me2 deposition is critical for preimplantation [34], we analyzed the H3K9me2 signal in zygotes. As shown in Fig. 1C, the signal intensity of H3K9me2 was increased in ERK1/2-inhibited paternal and maternal pronuclei compared to the controls (p < 0.01). Similar results were found in both MEK1/2 and p38-inhibited paternal and maternal pronuclei (p < 0.01). Meanwhile, the paternal/maternal signal ratio of H3K9me2 was greatly increased in ERK1/2-inhibited zygotes (p < 0.01), but decreased in both MEK1/2 and p38-inhibited zygotes (p < 0.01; Fig. 1C, D). Of note, H3K9me2 signal intensity was also increased in ERK1/2-inhibited paternal pronuclei when compared to that of MEK1/2 and p38-inhibited paternal pronuclei (p < 0.01).

The zygotes were further cultured 85 h after inhibition of ERK1/2 (Fig. 1E). As expected, the ERK1/2-inhibited embryos showed a decreased percentage of blastocysts in both 50 μM (60.48 ± 1.91% vs. 76.42 ± 1.39%, p < 0.01) and 100 μM GDC-0994 treatment group (39.05 ± 2.9% vs. 76.42 ± 1.39%, p < 0.01; Fig. 1F, G). Specifically, 100 μM GDC-0994 treatment led to an abnormality at the 2-, and 4-cell with a percentage of 16.61 ± 1.39% (p < 0.05) and 29.8 ± 0.936% (p < 0.01), respectively. Considering the effects of Oct4 and Nanog on lineage differentiation in the early embryo [35–39], we further investigated the transcription of several pluripotent genes in morula and blastocyst (Additional file 1: Fig. S3 and Fig. 1H). The transcription of *Oct4* (p < 0.05), *Nanog* (p < 0.05), *Sox2* (p < 0.05), and *Klf4* (p < 0.05) were all highly expressed in ERK1/2-inhibited blastocysts (Fig. 1H). Taken together, these data suggest that zygotic ERK1/2 is essential for embryo preimplantation development in mice.

### ERK1/2 regulates the H3K9me2 and DNA methylation reprogramming in paternal genomes

**Fig. 2:**
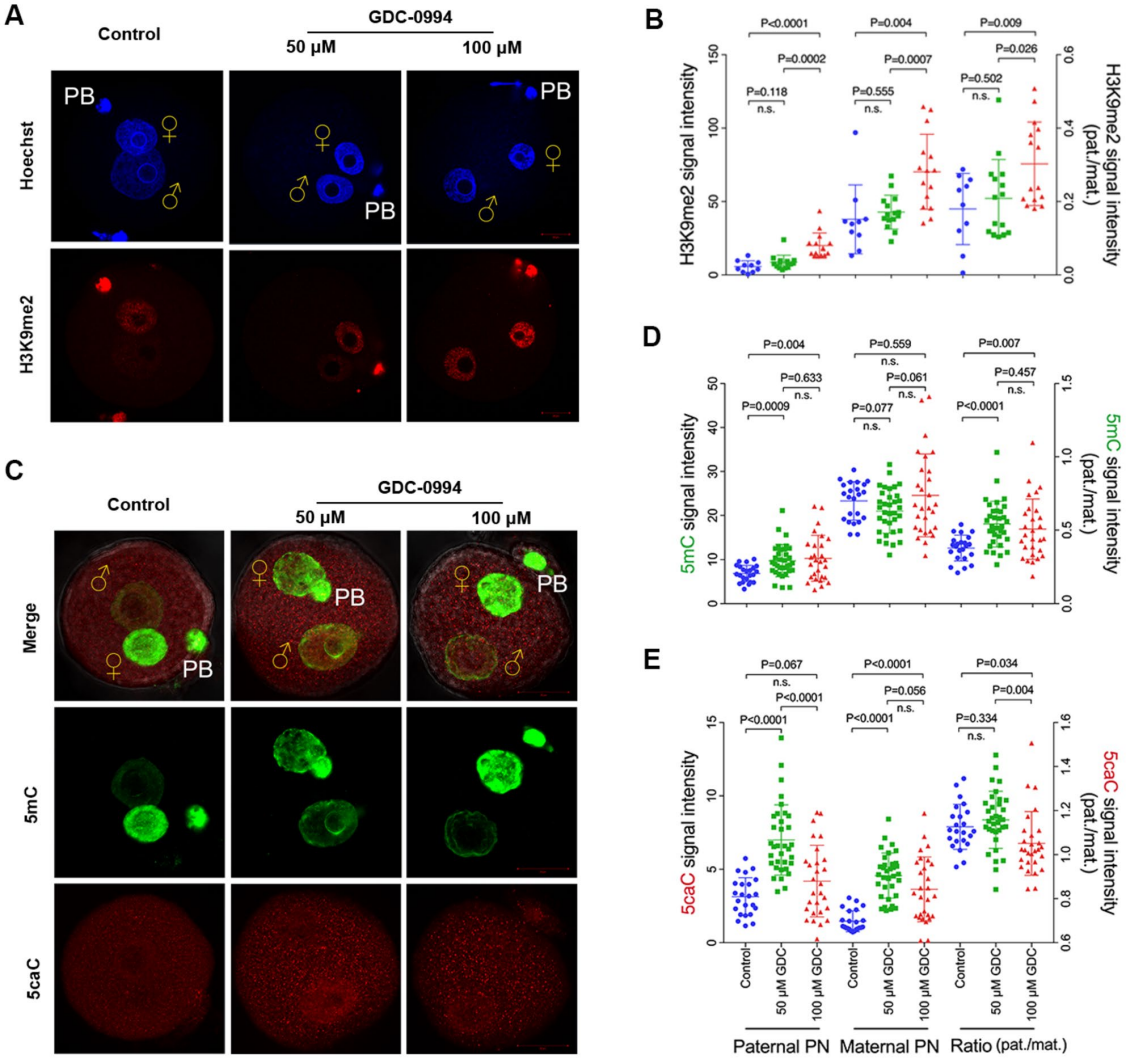
ERK1/2 regulates the H3K9me2 and DNA methylation reprogramming in paternal genomes. **A** H3K9me2 staining of control, 50 μM GDC-0994-treated, and 100 μM GDC-0994-treated zygotes at PN4-5 stage (10 hpf). **B** Values are represented as the mean of H3K9me2 signal intensity in paternal and maternal pronuclei after background subtraction (left axis) or a ratio between signals of parental pronuclei (pat./mat., right axis). Number of zygotes analysed for each group: control n = 10; 50 μM GDC-0094-treated n = 15; 100 μM GDC-0994-treated n = 15. **C** 5mC (green) and 5caC (red) staining of control, 50 μM GDC-0994-treated, and 100 μM GDC-0994-treated zygotes at PN4-5 stage (10 hpf). **D**, **E** Quantification of 5mC and 5caC is represented as signal intensity in paternal and maternal pronuclei (left axis) or as a ratio between parental signals (pat./mat., right axis). Number of zygotes analysed for each group: control n = 23; 50 μM GDC-0994-treated n = 34; 100 μM GDC-0994-treated n = 28. Statistical analysis was carried out using Student’s t-test (two-sided). P values are indicated. Error bars indicate SD. ♀, maternal pronucleus; ♂, paternal pronucleus. Scale bar, 20 µm

We next investigated whether the dynamic change of H3K9me2 is ERK1/2 dose-dependent. As shown in Fig. 2A, B, the signal intensity of H3K9me2 was not statistically changed in 50 μM GDC-0994 treatment zygotes compared to the controls. However, in 100 μM GDC-0994 treatment zygotes, the level of H3K9me2 signal was markedly increased in both the paternal and maternal pronuclei (p < 0.01), and the paternal to maternal signal ratio of H3K9me2 was also significantly increased (p < 0.01), suggesting that regulation of paternal H3K9me2 is ERK1/2 dose-dependent. We further examined the level of 5mC and 5caC signals in ERK1/2-inhibited zygotes. As shown in Fig. 2C, D, the level of paternal 5mC was increased following treatment with 50 μM GDC-0994 (p < 0.01). The product of the Tet3 oxidation chain, 5caC, was also accumulated more than the controls (p < 0.01) (Fig. 2C, E). Compared with the 50 μM GDC-0994 treated zygotes, the paternal 5mC remained stable in 100 μM GDC-0994-treated zygotes, but the accumulation of paternal 5caC was significantly reduced (p < 0.01), indicating that ERK1/2 might play pivotal roles in the oxidation of paternal 5mC. Notably, the asymmetries of 5mC, 5caC, and H3K9me2 between the paternal and maternal genomes were all decreased following 100 μM GDC-0994 treatment (p < 0.01, p < 0.05, p < 0.01, respectively) (Fig. 2A–E), suggesting that ERK1/2 is involved in the asymmetric regulation of these parental epigenetic marks in zygotes.

### ERK1/2 contributes H3K9me2 asymmetry by preventing paternal G9a localization

**Fig. 3:**
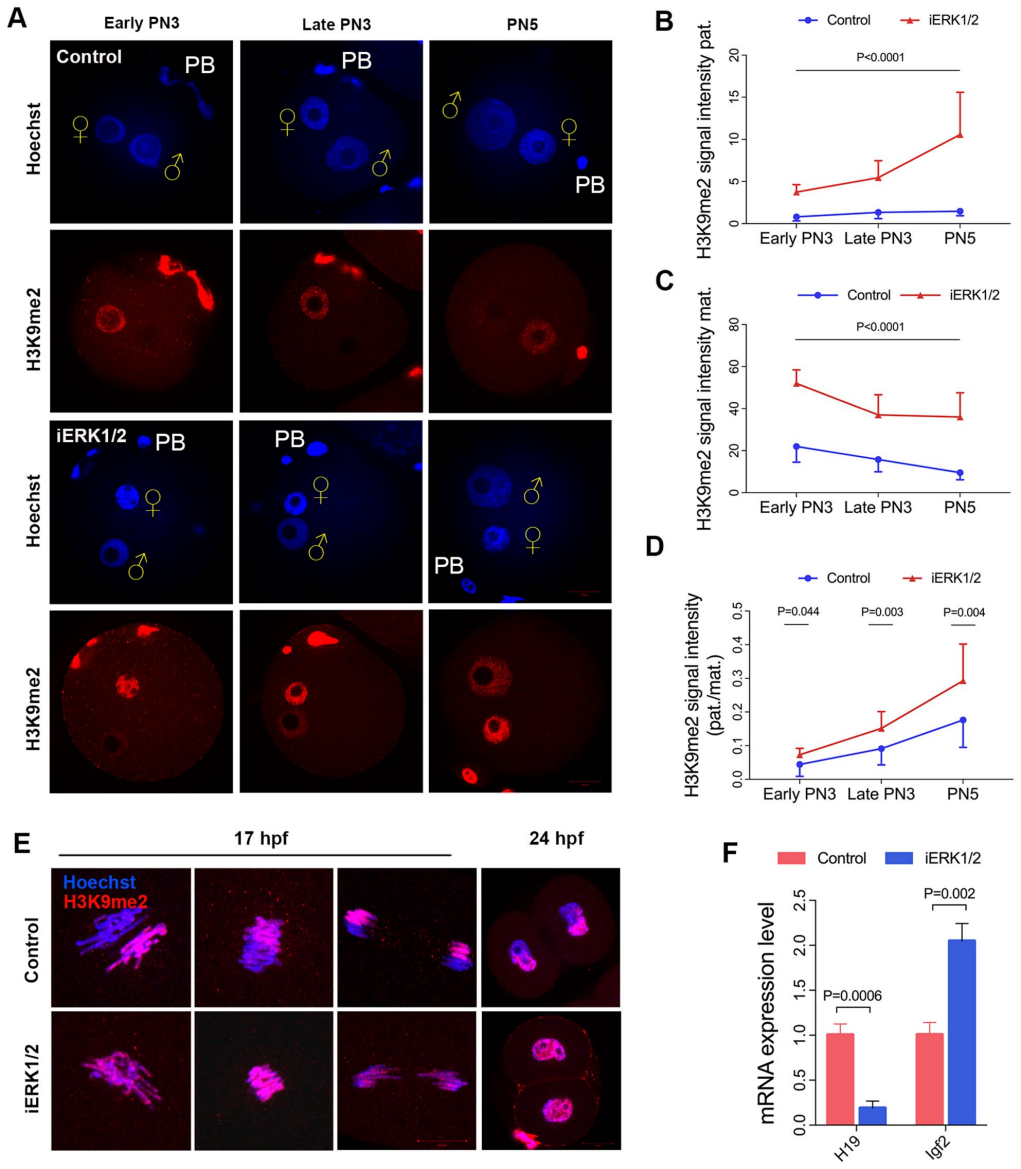
ERK1/2 impedes the deposition of paternal H3K9me2. **A** H3K9me2 deposition in ERK1/2-inhibited (100 μM GDC-0994) zygotes at different developmental stages. The pronuclear (PN) stages are indicated. Quantification of the H3K9me2 signal intensity in parental pronuclei (**B**, **C**) and the paternal to maternal signal ratio of H3K9me2 (**D**) in ERK1/2-inhibited zygotes. Number of zygotes analysed for each stage: Early PN3, n = 10; Late PN3, n = 11; PN5, n = 14 for control and Early PN3, n = 9; Late PN3, n = 19; PN5, n = 14 for GDC-0994-treatment. Each data point represents an independent zygote. **E** Representative images show equal distribution of H3K9me2 between paternal and maternal genomes in ERK1/2-inhibited (100 μM GDC-0994) 2-cell embryos compared to the controls. **F** The expression of *H19* and *Igf2* in ERK1/2-inhibited blastocysts. P values are indicated. Error bars indicate SD. ♀, maternal pronucleus; ♂, paternal pronucleus. PB, polar body. Scale bar, 20 µm

We reported the absence of paternal H3K9me2 in control zygotes in our previous studies [19, 20, 40]. However, paternal H3K9me2 showed pronuclear deposition with progressive enrichment since the early PN3 stage in ERK1/2-inhibited zygotes (Fig. 3A). The quantitative assessment revealed a significant and continuous deposition of paternal H3K9me2 (p < 0.01), while the maternal H3K9me2 increased rapidly and limitedly in ERK1/2-inhibited zygotes (p < 0.01) (Fig. 3B, C). The paternal to maternal signal ratio of H3K9me2 was greatly increased at the early PN3 (p < 0.05), late PN3 (p < 0.01), and PN5 (p < 0.01) in ERK1/2-inhibited zygotes compared to that of the controls (Fig. 3D), suggesting aberrant H3K9me2 deposition in minor ZGA when ERK1/2 was inhibited.

We further investigated the distribution of parental H3K9me2 at 17 hpf and 24 hpf. As shown in Fig. 3E, H3K9me2 was asymmetrically distributed at the 17 hpf and the 24 hpf in the controls. However, the asymmetry of parental H3K9me2 disappeared following treatment with GDC-0994 at the 17 and 24 hpf (Fig. 3E). H3K9me2 is important for protecting the methylation of paternally imprinted gene *H19* against active DNA demethylation [31]. The aberrant H3K9me2 asymmetry might impair the expression of *H19*. As expected, *H19* was significantly down-regulated whereas *Igf2* was highly expressed in blastocysts of the ERK1/2-inhibited group (Fig. 3F, p < 0.01, p < 0.01, respectively). Our data suggest that zygotic ERK1/2 is responsible for the asymmetry of parental H3K9me2 during ZGA by impeding the deposition of paternal H3K9me2.

**Fig. 4:**
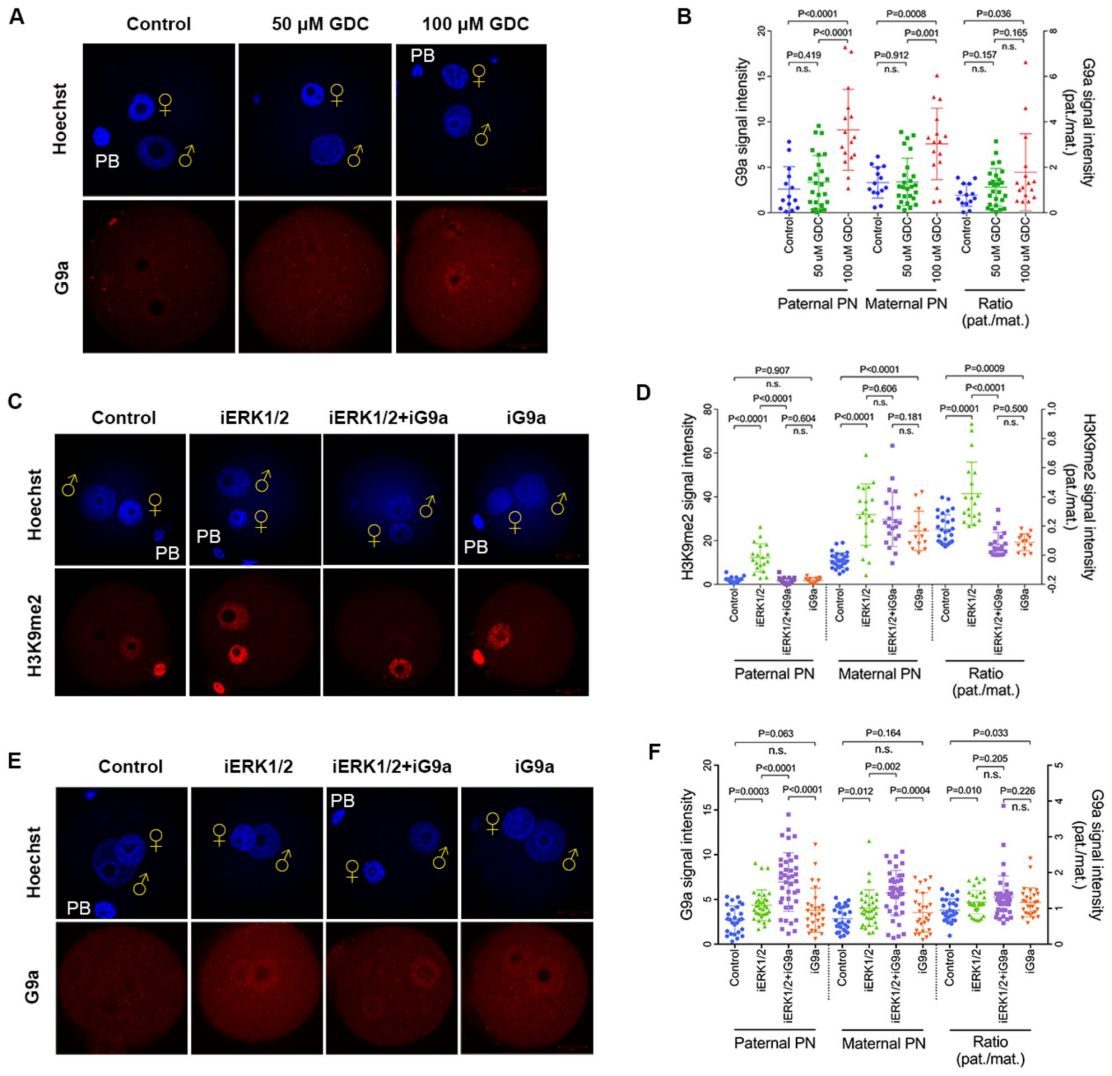
ERK1/2 contributes to the asymmetry of parental H3K9me2 by preventing paternal G9a localization. **A** G9a staining of control, 50 μM and 100 μM GDC-0994-treated zygotes at PN3 stage (5.5 hpf). **B** Quantification is represented as the mean of G9a signal intensity on parental pronuclei (left axis) or a ratio of G9a signal intensity between parental pronuclei (pat./mat., right axis). Number of zygotes analysed for each group: control n = 14; 50 μM GDC-0994-treated n = 26; 100 μM GDC-0994-treated n = 16. **C** Inhibition of G9a enzymes by BIX-01294 affects the deposition of paternal H3K9me2 at PN4-5 stage (10 hpf). **D** Quantification of the H3K9me2 signal intensity in parental pronuclei (left axis) or a ratio between the pronuclei signal (pat./mat., right axis). For H3K9me2 staining, control n = 29, iERK1/2 (100 μM GDC-0994) n = 19, iERK1/2 + iG9a n = 21; iG9a n = 14. **E** Under the same treatment conditions, G9a staining of control, iERK1/2 (100 μM GDC-0994), iERK1/2 + iG9a, and iG9a zygotes at PN4-5 stage (10 hpf). **F** Quantification of G9a staining in both paternal and maternal pronuclei (left axis) or a ratio between the pronuclear signals (pat./mat., right axis). For G9a staining, control n = 27, iERK1/2 n = 34, iERK1/2 + iG9a n = 40; iG9a n = 28. Statistical analysis was carried out using Student’s t-test (two-sided). P values are indicated. Error bars indicate SD. ♀, maternal pronucleus; ♂, paternal pronucleus. PB, polar body. Scale bar, 20 µm

Our previous study revealed that G9a is required for paternal H3K9me2 deposition in zygotes [21]. As shown in Fig. 4A, G9a was enriched in both paternal and maternal pronuclei (p < 0.01), and paternal to maternal signal ratio of G9a was increased following 100 μM (p < 0.05), but not 50 μM GDC-0994 treatment compared to the controls (Fig. 4A, B). To confirm that ERK1/2 regulates H3K9me2 asymmetry through G9a, we carried out IVF in the presence of BIX-01294, an inhibitor that competed for the substrate of G9a [41, 42]. Inhibition of G9a showed no statistical change of H3K9me2 in paternal pronuclei. However, the deposition of paternal H3K9me2 induced by ERK inhibition was barely detected in both ERK1/2 and G9a-inhibited zygotes, similar to the controls (Fig. 4C, D). In addition, the paternal pronuclear localization of G9a was not only increased in ERK1/2-inhibited zygotes, but also increased in both ERK1/2 and G9a-inhibited (GDC-0994 + BIX-01294-treated) zygotes (p < 0.01 Fig. 4E, F). These data demonstrate that ERK1/2 impedes paternal localization of G9a and contributes to the formation of H3K9me2 asymmetry between parental pronuclei.

### Inhibition of ERK1/2 promotes the oxidation of de novo 5mC in paternal pronuclei

**Fig. 5:**
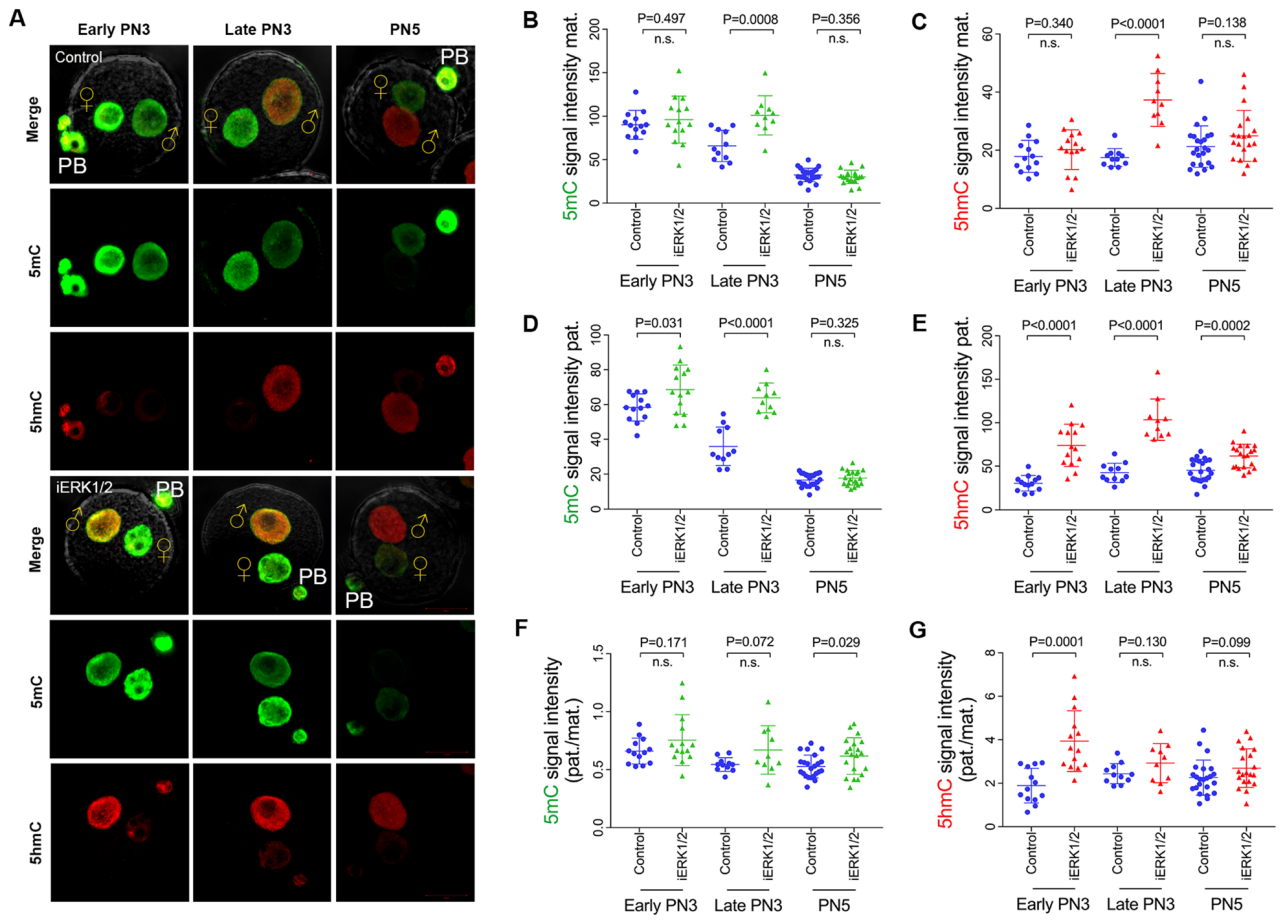
ERK1/2 Inhibition promotes the accumulation of 5mC and 5hmC in paternal pronuclei. **A** Representative immunofluorescence images of 5mC (green), 5hmC (red) in control and ERK1/2-inhibited (50 μM GDC-0994) zygotes at different stages. The pronuclear (PN) stages are indicated. Quantification is represented as the mean of 5mC and 5hmC signal intensity in parental pronuclei after background subtraction (**B**, **C**, mat. and **D**, **E**, pat.). **F**, **G** Quantification of 5mC (left) and 5hmC (right) staining as a ratio between the pronuclear signals (pat/mat). Number of zygotes analyzed for each stage: Early PN3, 13; Late PN3, 11; PN5, 23 for control and Early PN3, 14; Late PN3, 10; PN5, 20 for GDC-0994. Each data point represents a zygote. P values are indicated. Error bars indicate SD. ♀, maternal pronucleus; ♂, paternal pronucleus. PB, polar body. Scale bar, 20 µm

Since 5mC and 5caC were both accumulated in paternal pronuclei with 50 μM GDC-0994 treatment, we next assessed the dynamic changes of 5hmC in parental pronuclei following 50 μM GDC-0994 treatment. As shown in Fig. 5A–C, both 5mC and 5hmC were increased at the late PN3 in maternal pronuclei with 50 μM GDC-0994 treatment (p < 0.01). In paternal pronuclei, the 5mC signal intensity was increased at the early PN3 (p < 0.05) and late PN3 (p < 0.01), meanwhile, 5hmC level was higher in early PN3, late PN3, and PN5 stage in ERK1/2-inhibited zygotes (p < 0.01) (Fig. 5D, E), suggesting that inhibition of ERK1/2 promotes oxidation of paternal 5mC. Moreover, the paternal to maternal signal ratios of 5mC (p < 0.05) and 5hmC (p < 0.01) were increased at the PN5 stage and the early PN3, respectively (Fig. 5F, G).

**Fig. 6:**
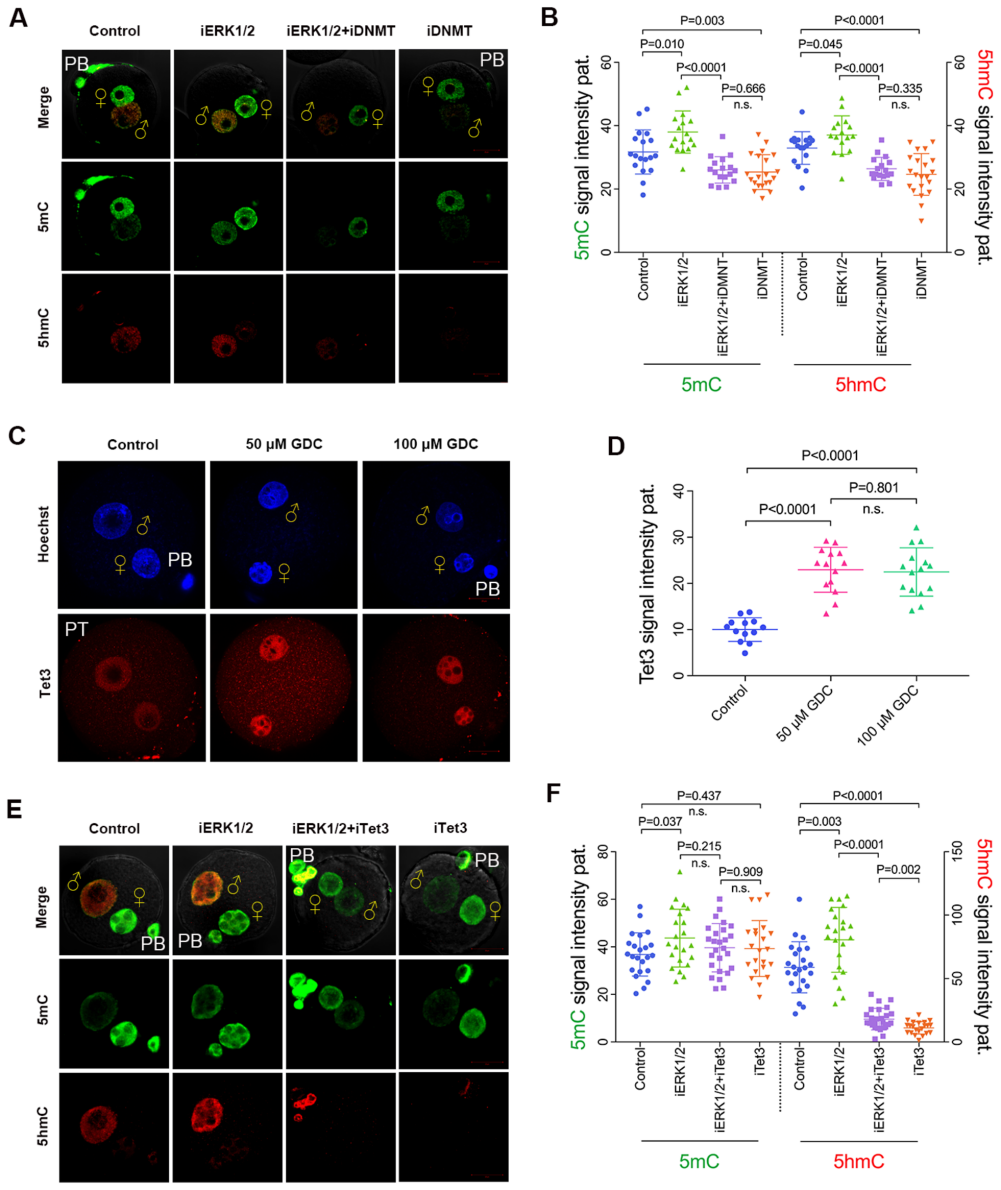
ERK1/2 inhibition promotes Tet3-driven oxidation of de novo 5mC in paternal pronuclei. **A** Impediment of de novo 5mC by Decitabine affects paternal 5hmC accumulation in ERK1/2-inhibited zygotes (10 hpf) as assessed by 5mC and 5hmC staining. **B** Quantification of 5mC (left axis) and 5hmC (right axis) is represented as the signal intensity in paternal pronuclei (n = 18 for control, n = 17 for iERK1/2, n = 17 for iERK1/2 + iDNMT; n = 21 for iDNMT). **C** Tet3 staining under PT conditions of the control, 50 μM GDC-0094-treated, and 100 μM GDC-0994-treated zygotes at PN3 stage (5.5 hpf). **D** Quantification of Tet3 is represented as signal intensity in paternal pronuclei after background subtraction. Number of zygotes analysed for each group: control n = 13, 50 μM GDC-0994-treated n = 14, 100 μM GDC-0994-treated n = 15. **E** Inhibition of Tet enzymes by DMOG impedes the paternal 5hmC accumulation ERK inhibition produced (50 μM GDC). Representative images of 5hmC and 5mC staining at PN4-5 (10 hpf). **F** Quantification of 5mC (left axis) and 5hmC (left axis) is represented as signal intensity in paternal pronuclei (n = 23 for control, n = 22 for iERK1/2, n = 26 for iERK1/2 + iTet3; n = 22 for iTet3). Statistical analysis was carried out using Student’s t-test (two-sided). P values are indicated. Error bars indicate SD. ♀, maternal pronucleus; ♂, paternal pronucleus. PB, polar body. Scale bar, 20 µm

**Fig. 7:**
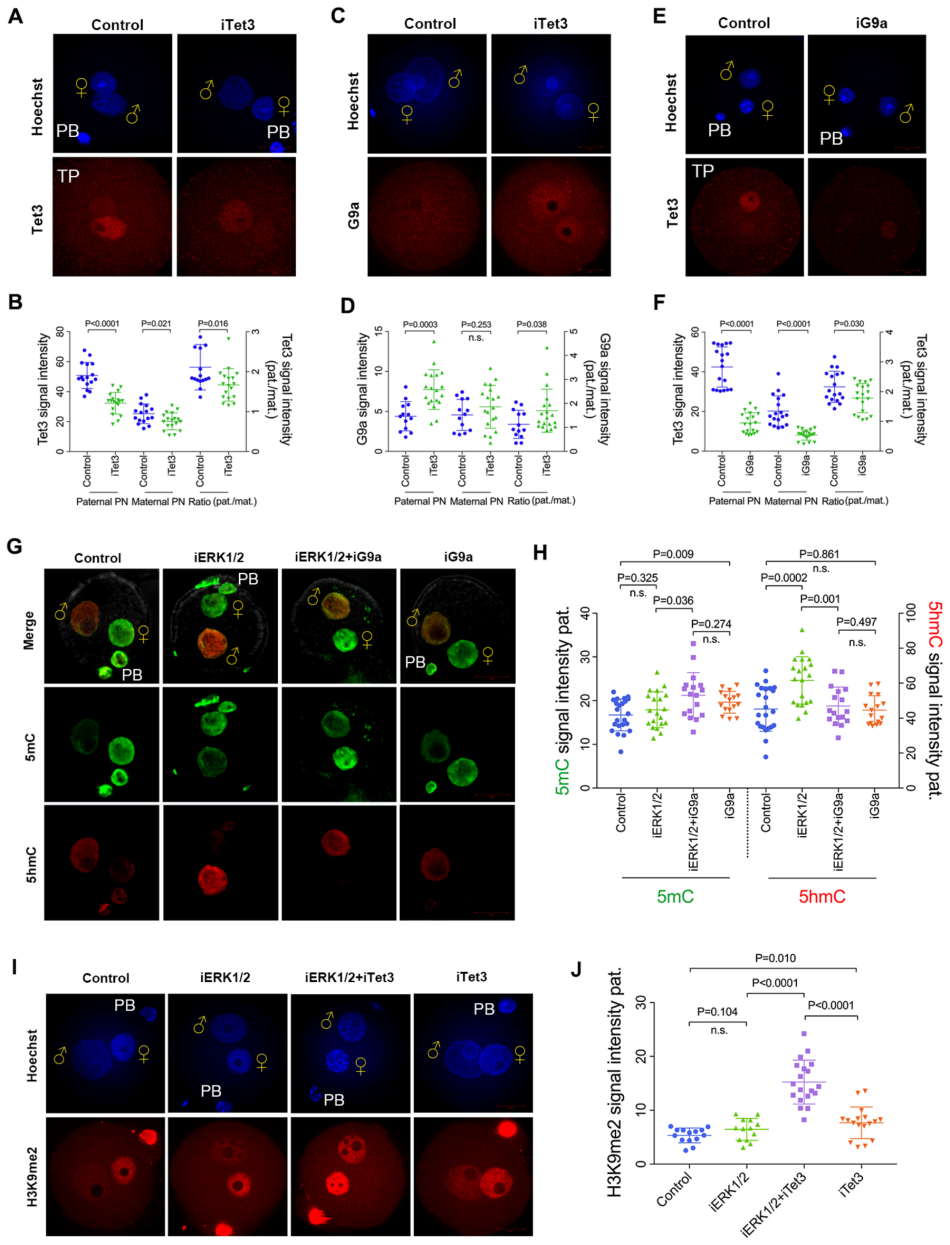
Excess pronuclear localization of G9a impedes 5mC oxidation in paternal pronuclei. **A** Tet3 staining under TP conditions of control and DMOG-treated zygotes at the PN4-5 stage (10 hpf). **B** Quantification is represented as Tet3 signal intensity or a ratio between parental signals (pat./mat.). (n = 16 for control, n = 18 for iTet3.). **C** G9a staining of control, and DMOG-treated zygotes at the PN4-5 stage (10 hpf). **D** Quantification is represented as G9a signal intensity or a ratio (pat/mat). (n = 13 for control, n = 19 for iTet3). **E** Tet3 staining under TP conditions of control, and BIX-01294-treated zygotes at the PN4-5 stage (10 hpf). **F** Quantification is represented as Tet3 signal intensity or a ratio (pat./mat.) (n = 18 for control, n = 19 for iG9a.) **G** Competitive inhibition of G9a activity by BIX-01294 affects the accumulation of paternal 5hmC ERK1/2 inhibition (50 μM GDC-0994) produced as assessed by 5mC and 5hmC staining at the PN5 stage (11 hpf). **H** Quantification of 5mC and 5hmC is represented as signal intensity in paternal pronuclei. (n = 23 for control, n = 20 for iERK1/2, n = 17 for iERK1/2 + iG9a; n = 16 for iG9a.) **I** Tet3 inhibition by DMOG promotes the deposition of paternal H3K9me2 as assessed by H3K9me2 staining at PN4-5 stage (10hpf). **J** Quantification of the parental H3K9me2 signal intensity. Number of zygotes analysed for each group: n = 14 for control, n = 13 for iERK1/2, n = 19 for iERK1/2 + iTet3; n = 17 for iTet3. Statistical analysis was carried out using Student’s t-test (two-sided). P values are indicated. Error bars indicate SD. ♀, maternal pronucleus; ♂, paternal pronucleus. PB, polar body. Scale bar, 20 µm

We further investigated the roles of DNMT and Tet3 in the yields of paternal 5mC and 5hmC when ERK1/2 was inhibited. Decitabine and DMOG were used for the inhibition of DNMT and Tet3 in zygotes as described previously [30], and the suppression effect has been verified (Additional file 1: Fig. S4 and Fig. 7A, B). As shown in Fig. 6A, B, paternal 5mC level was reduced in both ERK1/2 and DNMT-inhibited zygotes, as well as in DNMT-inhibited zygotes compared with that of ERK1/2 inhibition (p < 0.01). Interestingly, the localization of Tet3 in paternal pronuclei was increased when ERK1/2 was inhibited (p < 0.01) (Fig. 6C, D). However, the 5mC level was not affected by Tet3 inhibition (Fig. 6E, F). These data indicate that inhibition of ERK1/2 promotes the accumulation of de novo 5mC in paternal pronuclei.

In the absence of de novo 5mC in paternal pronuclei, ERK1/2 inhibition induced 5hmC was eliminated in both ERK1/2 and DNMT-inhibited zygotes (p < 0.01) (Fig. 6A, B), suggesting that DNMT-driven de novo 5mC is required for the excess accumulation of paternal 5hmC in ERK1/2-inhibited zygotes. We further investigated whether the excess paternal 5hmC in ERK1/2-inhibited zygotes was produced by Tet3. As shown in Fig. 6E, F, the excess accumulation of paternal 5hmC induced by ERK1/2 inhibition disappeared in both ERK1/2 and Tet3-inhibited zygotes (p < 0.01). Taken together, our results demonstrate that ERK1/2 inhibition promotes Tet3-driven oxidation of excess de novo 5mC in paternal pronuclei, and subsequently leads to accumulation of paternal 5hmC.

### Excess localization of G9a impedes oxidation of 5mC in paternal pronuclei

Since the pronuclear localization of G9a and Tet3 was both increased after ERK1/2 inhibition, and the GDC-0994 concentration required for accumulation of paternal Tet3 was lower than that of paternal G9a, we further assessed the interaction between G9a and Tet3. Under the TP conditions, we found reduced tight binding of Tet3 to chromatin following DMOG treatment, especially in paternal pronuclei (p < 0.01) (Fig. 7A, B). Conversely, the G9a localization was increased significantly in paternal pronuclei (p < 0.01), but not in maternal pronuclei with DMOG treatment (Fig. 7C, D). Moreover, paternal Tet3 was decreased in G9a-inhibited (BIX-01294-treated) zygotes (p < 0.01) (Fig. 7E, F). These data suggest that the accumulation of pronuclear G9a resulted in the reduction of Tet3 localization in paternal pronuclei.

To validate the interaction between Tet3 and G9a, we detected the level of paternal 5mC and 5hmC signals in the presence of both ERK1/2 and Tet3 inhibitors. As shown in Fig. 7G, H, both inhibition of ERK1/2 and G9a catalyst activity (50 μM GDC-0994 and BIX-01294, respectively) resulted in the accumulation of paternal 5mC (p < 0.05) and the loss of paternal 5hmC (p < 0.01) compared to the ERK1/2-inhibited zygotes. Since inhibition of both ERK1/2 and G9a led to a loss of H3K9me2 in paternal pronuclei, these data suggest that excess localization of G9a impedes oxidation of 5mC in paternal pronuclei by inhibition of Tet3. Moreover, paternal H3K9me2 was increased in both ERK1/2 and Tet3-inhibited (50 μM GDC-0994 and DMOG, respectively) zygotes (Fig. 7I, J), suggesting that Tet3 impedes the paternal H3K9me2 deposition in zygotes as well.

## Discussion

In the present study, we found that fertilization-dependent ERK1/2 is the critical regulator for early embryo development in mice. We further reported reduced asymmetries of 5mC, 5hmC, 5caC, and H3K9me2 at the PN stage after ERK1/2 inhibition. In addition, ERK1/2 inhibition led to excess pronuclear localization of G9a, which subsequently antagonizes the tight binding of Tet3 to paternal chromatin, providing crucial insights into the regulation of DNA methylation and histone modifications crosstalk by ERK1/2.

Previous studies revealed that ERK1/2 protein levels increase after fertilization [14, 43, 44], which is the minor ZGA. Consistently, we found that ERK1/2 was highly expressed during ZGA, as revealed by RNA sequencing data. We further observed that multiple MAPKs pathways are involved in the regulation of the maternal H3K9me2 deposition. As for paternal genomes, H3K9me2 deposition showed the most significant increase, and the asymmetry of the parental H3K9me2 was altered following zygotic ERK1/2 inhibition, indicating that ERK1/2 plays an important role in the regulation of paternal H3K9me2 and the asymmetry between the parental genomes.

It was demonstrated that mouse minor ZGA occurs at the PN3 stage, during which, the first zygotic transcription is promiscuous [45] and the epigenetic modifications are rapidly reprogrammed [5, 30, 31, 46]. In the present study, we found decreased levels of 5mC and H3K9me2 in maternal pronuclei from the PN3 stage, consistent with previous studies [21, 47, 48]. Considering that 5mC and H3K9me2 are transcriptional repressive markers [24, 25, 49, 50], the removal of 5mC and H3K9me2 would be important for the initiation of minor ZGA. Indeed, studies reported the symmetry distribution of 5mC between paternal and maternal pronuclei at PN stage impairs the embryonic development [32] and that of H3K9me2 is embryonic lethal [22], suggesting the asymmetries distribution of 5mC and H3K9me2 are critical for the early embryo development.

A recent study revealed that ERK is related to transcription activity and the fluctuation or persistence of ERK signal would irreversibly change the expression of pluripotency genes [51]. In the present study, the expression of two genes *Oct4* and *Nanog*, which serve as hubs in the core pluripotency network, was increased in the expanded and hatched blastocysts after zygotic ERK1/2 inhibition, suggesting that the regulation of ERK on the determination of the embryonic fate occurs in the first cell cycle, consistent with the results of directing fate specification in the preimplantation embryo by ERK [15].

The level of the 5hmC signal was increased in both paternal and maternal pronuclei at the PN3 stage following the treatment with a low concentration of ERK1/2 inhibitor. A previous study reported the suppression of Tet1 by the ERK pathway in cells [52]. However, Tet1 was barely expressed at the PN stage. Therefore, we focused on Tet3, which has previously been reported as a key regulator of the 5hmC asymmetry between paternal and maternal pronuclei [30, 31, 53]. As expected, the Tet3 localization in paternal pronuclei was increased following the treatment with a low concentration of ERK1/2 inhibitor, suggesting that ERK1/2 controls the process of paternal 5mC oxidation by preventing pronuclear localization of Tet3 in the zygotic genome.

G9a is essential for preimplantation development [34, 54] and limits the range of the promiscuous transcriptions during exposure to stress [55]. Following the treatment with a higher concentration of ERK1/2 inhibitor, the H3K9me2 deposition and G9a localization in paternal pronuclei were both increased. Meanwhile, the number of blastocysts from zygotes with the same treatment was decreased sharply, suggesting that the limitation of G9a on the promiscuous transcription of the zygotic genome and the lethality of excess H3K9me2 deposition in paternal pronuclei during early development.

Our study shows that G9a plays a vital role in 5mC oxidation during paternal genomic reprogramming. The excess pronuclear localization of G9a antagonized the tight binding of Tet3 to paternal chromatin and effectively blocked the accumulation of paternal 5hmC in both ERK1/2 and G9a-inhibited zygotes, suggesting that fertilization-dependent ERK1/2 promotes rapid oxidation of paternal 5mC by inhibiting G9a pronuclear localization. The catalytic activity of G9a has previously been reported that essential for protecting the maternal genomic methylation from Tet3-mediated 5mC oxidation [22]. Interestingly, in both ERK1/2 and Tet3-inhibited zygotes where G9a pronuclear localization increased and catalytic activity was maintained, the paternal genomes showed a significant H3K9me2 deposition and a downward trend in de novo 5mC accumulation that ERK1/2 inhibition produced. Furthermore, compared with paternal genomes, the accumulation of de novo 5mC and 5hmC in maternal genomes both showed a lag after ERK1/2 inhibition. These data are consistent with the notion that H3K9me2 protects the zygotic genome against the de novo 5mC [56, 57]. Therefore, our findings support the notion that G9a pronuclear localization impedes the oxidation of paternal 5mC during zygotic epigenetic reprogramming, regardless of whether G9a retains its catalytic performance or not.

Our study also reveals the crosstalk between 5mC and H3K9me2 in the zygotic genome. In our ERK1/2 inhibition system, Tet3 converts de novo 5mC into 5hmC, while the deposition of H3K9me2 prevents the accumulation of de novo 5mC in the zygotic genome. Recently, the particular epigenetic landscapes show that 5mC is largely independent of H3K9me2 in differentiated [33] and diseased cells [58, 59]. H3K9me2 protects 5mC inherited from gametes, but it might prevent the accumulation of de novo 5mC at some loci in zygotes. Therefore, this independence between genomic 5mC and H3K9me2 can be explained by DNA replication and the inheritance 5mC dilution.

## Conclusions

We have demonstrated that the inhibition of zygotic ERK1/2 causes excess deposition of H3K9me2 and accumulation of 5mC and its oxides in paternal pronuclei by triggering the pronuclear localization of G9a and Tet3, respectively. In conclusion, we propose that zygotic reprogramming is regulated by fertilization-dependent ERK1/2, which contributes to the development competence of pre-implantation embryos in mice.

## Materials and methods

### Sperm collection

Sperm was obtained from ICR males aged 10–20 weeks. The cauda epididymis was cut open with the tip of syringes to allow sperm swimming out. A mass of sperm was put into Human Tubal Fluid (HTF) fertilization medium supplemented with 4 mg/ml bovine serum albumin (BSA, Sigma-Aldrich) and incubated for 1–1.5 h at 37 °C in 5% CO_2_.

### In vitro fertilization

ICR females aged 4–6 weeks were superovulated by intraperitoneal injection of 10 U pregnant mare’s serum (PMS) and 10 U of human chorionic gonadotropin (HCG) 48 h later. Cumulus oocyte complexes collected 14 h post HCG injection were incubated with capacitated sperm for 2.5 h. Tet3 inhibition was performed by supplementing with 1 mM dimethyloxallyl glycine (DMOG, Sigma-Aldrich) in fertilization medium; oocytes were incubated with DMOG for at least 40 min before addition of sperm.

### Zygotes culture and collection

Zygotes were washed off the excess sperm and granulosa cells at 2.5 hpf, and were randomly divided into several equal parts for different treatments: GDC-0994 (50–100 μM, Selleck, #s7554), U0126-EtOH (40 μM, Selleck, #s1102), SB203580 (100 μM, Selleck, #s1076), SP600125 (50–100 μM, Selleck, #s1460), BIX-01294 (10 μM, Selleck, #s8006), DMOG (1 mM, Sigma, #D3695), or Decitabine (10 μM, Selleck, #s1200), and collected at times indicated. Zygotes incubated with the matching concentration of dimethylsulphoxide (DMSO, 0.1–0.2%) were served as the controls. Zygotes at early and late PN3, PN5 stages, mitotic phase 1- and 2-cell embryos were collected at 5.5, 8, 11, 17, and 24 hpf, respectively. For preimplantation embryos culture, zygotes were incubated with GDC-0994 for 8.5 h and subsequently transferred into KSOM medium without GDC-0994 for further culture to embryonic day E4.

### Immunofluorescence staining

Zygotes were fixed in 3.7% paraformaldehyde (PFA) for 60 min, and permeabilized in PBS, 1% BSA, 0.5% Triton X-100 for 25 min at room temperature (RT). Zygotes were blocked for 1 h in PBS, 1% BSA at RT and incubated with the following primary antibody: H3K9me2 (Cell Signaling Technology, #4658, 1:100), G9a (Cell Signaling Technology, #3306, 1:100), and Tet3 (Abcam, #ab153724, 1:200) overnight at 4 °C. Zygotes were then incubated with Alexa Fluor 488- or 555-conjugated IgG secondary antibody (Molecular Probes, 1:200) for 1 h in dark. DNA was stained by 1 μg/ml Hoechst33342 (Beyotime, Shanghai, China) for 15 min, and zygotes were mounted in Vectashield (Vector laboratories) and were visualized using an LSM710 confocal laser scanning microscope (Carl Zeiss) with a × 40 objective.

TP conditions are used for Tet3 staining in zygotes. Triton pre-extraction was performed as previously described with minor modifications [30, 31]. Briefly, zygotes were incubated in ice-cold permeabilization solution (50 mM NaCl, 3 mM MgCl_2_, 0.5% Triton X-100, 300 mM sucrose in 25 mM HEPES pH 7.4) for 45–60 s until the perivitelline space was eliminated, followed by PFA fixation.

### Immunofluorescence staining of 5mC, 5hmC, and 5caC

After PFA fixation and permeabilization, zygotes were treated with 4 M HCl for 10 min at RT. Zygotes were then incubated in Tris–HCl 100 mM for 10 min, and washed in PBS, 1% BSA, 0.05% Tween20 three times for 15 min. The rinsed zygotes were blocked for 1 h in 1% BSA, 0.05% Tween20 in PBS at RT. Zygotes were then incubated with 5mC (Millipore, #NA81-50UG, 1:500), 5hmC (Active Motif, #39769, 1:500) or 5caC (diagenode, # C15410204, 1:500) antibody overnight at 4 °C, and subsequently incubated with Alexa Fluor 488- or 555-conjugated IgG secondary antibodies (1:200) for 1 h in dark at RT. DNA was stained by 1 μg/ml Hoechst33342 for 15 min, and zygotes were mounted in Vectashield and imaged as described above.

### Western blotting analysis

MEK1/2 (Cell Signaling Technology, #8727, 1:1000), phospho-MEK1/2 (Cell Signaling Technology, #9154, 1:1000), ERK1/2 (Cell Signaling Technology, #4695, 1:1000), phospho-ERK1/2 (Cell Signaling Technology, #4370, 1:1000), p38 (Cell Signaling Technology, #8690, 1:1000), phospho-p38 (Cell Signaling Technology, #4511, 1:1000), SAPK/JNK (Cell Signaling Technology, #9252, 1:1000), phospho-SAPK/JNK (Cell Signaling Technology, #4668, 1:1000), Dnmt1 (Abcam, ab188453, 1:1000), Dnmt3a (Abcam, ab188470, 1:2000), and beta Actin (Bioss, bs-0061R, 1:1000) protein expression in zygotes were verified by western blot analysis. 48–150 mouse zygotes per group were placed in ice-cold RIPA lysis buffer (Beyotime, Shanghai, China) containing 1% PMSF (Beyotime, Shanghai, China). The protein was added with 5× SDS-PAGE Sample Loading Buffer (Beyotime, Shanghai, China) and then denatured on a PCR instrument at 100 °C for 10 min. Proteins were separated by SDS-PAGE (GenScript) at 130 V for 60 min and then electrophoretically transferred to polyvinylidene fluoride (PVDF) membranes (Merck, Millipore, Ltd). After transfer, membranes were blocked with TBST that contained 5% BSA for 2 h, followed by incubation at 4 °C overnight with a rabbit primary antibody. After washing three times in TBST (5 min each), membranes were incubated with Horseradish peroxidase-labeled anti-rabbit IgG secondary antibody (dilution 1:5000; Cell Signaling Technology) for 2 h at RT. Finally, the specific proteins were visualized using Western blotting detection kit (Advansta) and analyzed by ImageJ software. After detection of phosphorylated antigen, the membrane was incubated with membrane regeneration solution (Solarbio) for 60 min for stripping and reprobing of the immunoblot of the total protein levels in the same sample.

### Gene expression analysis

RNA-seq data of mice (GSE98150) were downloaded from Gene Expression Omnibus. Gene expression of MAPKs during early embryo development was normalized with log2(count + 1).

Blastocysts (30 for each treatment) were collected at day E4, and mRNA was purified using Dynabeads mRNA DIRECT™ KIT (invitrogen) following the manufacturer’s instructions. Random primed reverse transcription was performed using HiScript III RT SuperMix with gDNA wiper (R323-01, Vazyme, Nanjing, China). cDNA was added to 10 µl of quantitative PCR mix (Q111-02, Vazyme, Nanjing, China). RT-PCR reactions were performed on a Step-One Plus Real-Time PCR system (Applied Biosystems, Carlsbad, CA, USA). The primers for quantitative analysis are shown in Additional file 2: Table S1. Gene expression was calculated using the 2^ΔΔct^ method, and *GAPDH* was used for normalization as endogenous reference genes.

### Data analysis

Images were analyzed using ImageJ software. The midsection of each pronucleus was identified using Hoechst33342 staining and determined by the maximal area. The midsection was used to quantify the total intensity following the subtraction of the signal corresponding to the cytoplasmic area (representing staining background). Statistical analysis was carried out using two-tailed Student’s t-test with Welch’s correction when required, using GraphPad Prism software. For gene expression analysis, statistical analysis was performed using two-tailed unpaired t-test. At least three biological replicates were performed for each analysis. Each replicate was conducted by an independent experiment at different times.

## Supplementary Information

**Additional file 1: Figure S1.** Phenotype of  zygotes following treatment with inhibitors of MEK1/2, ERK1/2, p38, and JNK. **Figure S2.** The expression of phosphorylated and total MAPKs proteins in zygotes treated with corresponding inhibitors. **Figure S3.** The expression of pluripotent genes in morula that zygotic ERK1/2 inhibited. **Figure S4.** The expression of Dnmt1 and Dnmt3a in DNMT-inhibited zygotes.

**Additional file 2: Table S1.** Primers used for qRT-PCR analysis.

## Data Availability

The datasets used and analyzed during the current study are available from the corresponding author on reasonable request.
