# Supplementary material for: Regulation of paternal 5mC oxidation and H3K9me2 asymmetry by ERK1/2 in mouse zygotes
Source: Cell Biosci. 2022 Mar 7;12:25. doi: 10.1186/s13578-022-00758-x (PMC8900417; doi:10.1186/s13578-022-00758-x)
Supplement: Supplementary file 1 — Additional file 1: Figure S1. Phenotype of zygotes following treatment with inhibitors of MEK1/2, ERK1/2, p38, and JNK. Figure S2. The expression of phosphorylated and total MAPKs proteins in zygotes treated with corresponding inhibitors. Figure S3. The expression of pluripotent genes in morula that zygotic ERK1/2 inhibited. Figure S4. The expression of Dnmt1 and Dnmt3a in DNMT-inhibited zygotes. [file 13578_2022_758_MOESM1_ESM.docx]

**Additional file1 Figure S1**

**
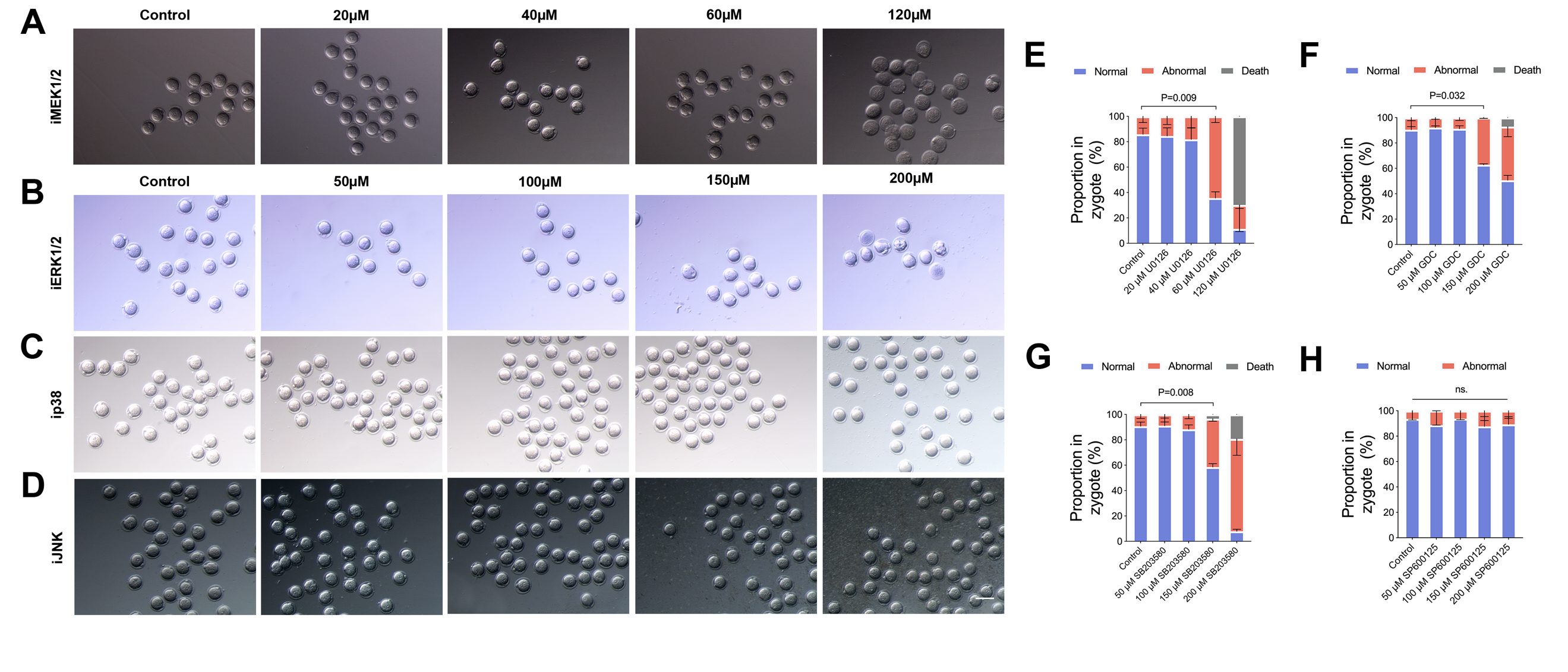
**

**Additional file1: Figure S1.** Phenotype of zygotes following treatment with inhibitors of MEK1/2, ERK1/2, p38, and JNK. (**A**-**D**) Bright-field images revealed morphological changes of zygotes treated with different inhibitors (U0126 for iMEK1/2, GDC-0994 for iERK1/2, SB203580 for ip38, and SP600125 for iJNK, respectively.) at PN4-5 stage (10hpf). Scale bars, 100 µm. (**E**-**H**) Percentage of abnormal zygotes after U0126, GDC-0994, SB203580, and SP600125 treatment. Total number of zygotes analysed: iMEK1/2, n =237 for Control, n=235 for 20 μM U0126 treatment, n=238 for 40 μM U0126 treatment, n=138 for 60 μM U0126 treatment, n=138 for 120 μM U0126 treatment; iERK1/2, n =203 for Control, n=203 for 50 μM GDC-0994 treatment, n=202 for 100 μM GDC-0994 treatment, n=78 for 150 μM GDC-0994 treatment, n=78 for 200 μM GDC-0994 treatment; ip38, n =173 for Control, n=173 for 50 μM SB203580 treatment, n=171 for 100 μM SB203580 treatment, n=73 for 150 μM SB203580 treatment, n=74 for 200 μM SB203580 treatment; iJNK, n =210 for Control, n=212 for 50 μM SP600125 treatment, n=212 for 100 μM SP600125 treatment, n=135 for 150 μM SP600125 treatment, n=137 for 200 μM SP600125 treatment. Statistical analysis was carried out using Student’s t-test (two-sided). Error bars indicate SD. P values are indicated.

**Additional file1 Figure S2**

**
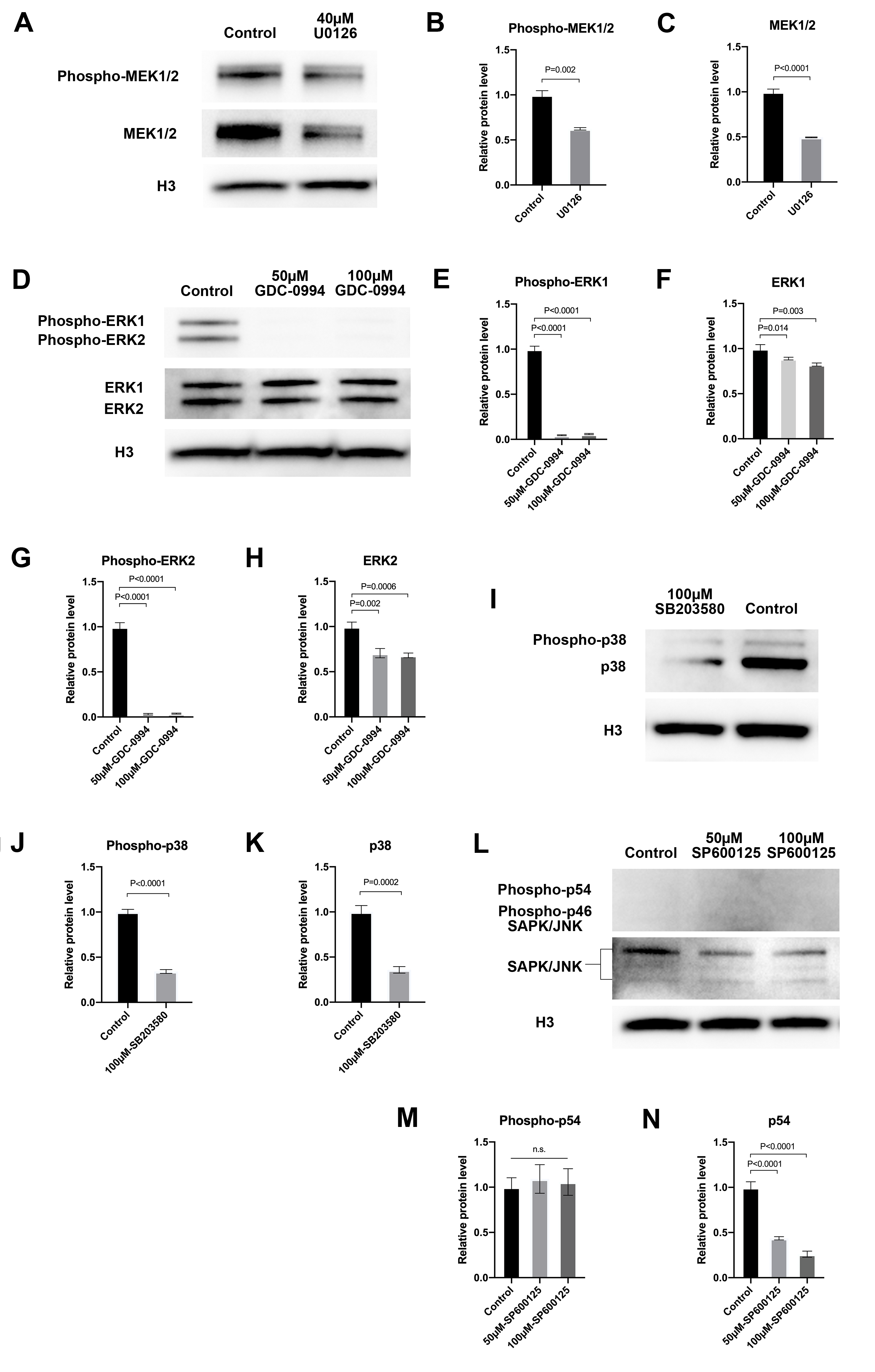
**

**Additional file1: Figure S2.** The expression of phosphorylated and total MAPKs proteins in zygotes treated with corresponding inhibitors. **A** Phospho-MEK1/2 and MEK1/2 proteins expression in zygotes (10 hpf) following treatment with 40μM U0126 were verified by western blot analysis. Number of zygotes analysed for each group: control n=112; U0126-treated n=112. Statistical analysis of phospho-MEK1/2 (**B**) and MEK1/2 (**C**) in control and U0126-treated zygotes. **D** Phospho-ERK1/2 and ERK1/2 proteins expression in zygotes (10 hpf) following treatment with GDC-0994 were verified by western blot analysis. Number of zygotes analysed for each group: control n=48; 50μM GDC-0994-treated n=48; 100μM GDC-0994-treated n=48. Statistical analysis of phospho-ERK1/2 (**E**, **G**) and ERK1/2 (**F**, **H**) in control and GDC-0994-treated zygotes. **I** Phosphor-p38 and p38 proteins expression in zygotes (10 hpf) following treatment with 100μM SB203580 were verified by western blot analysis. Number of zygotes analysed for each group: control n=128; SB203580-treated n=128. Statistical analysis of phospho-p38 (**J**) and p38 (**K**) in control and SB203580-treated zygotes. **L** Phospho-SAPK/JNK and SAPK/JNK proteins expression in zygotes (10 hpf) following treatment with SP600125 were verified by western blot analysis. Number of zygotes analysed for each group: control n=150; 50μM SP60025-treated n=150; 100μM SP600125-treated n=150. Statistical analysis of phospho-p54 (**M**) and p54 (**N**) in control and SP600125-treated zygotes. Statistical analysis was carried out using Student’s t-test (two-sided). Error bars indicate SD. P values are indicated.

**Additional file1 Figure S3**

**
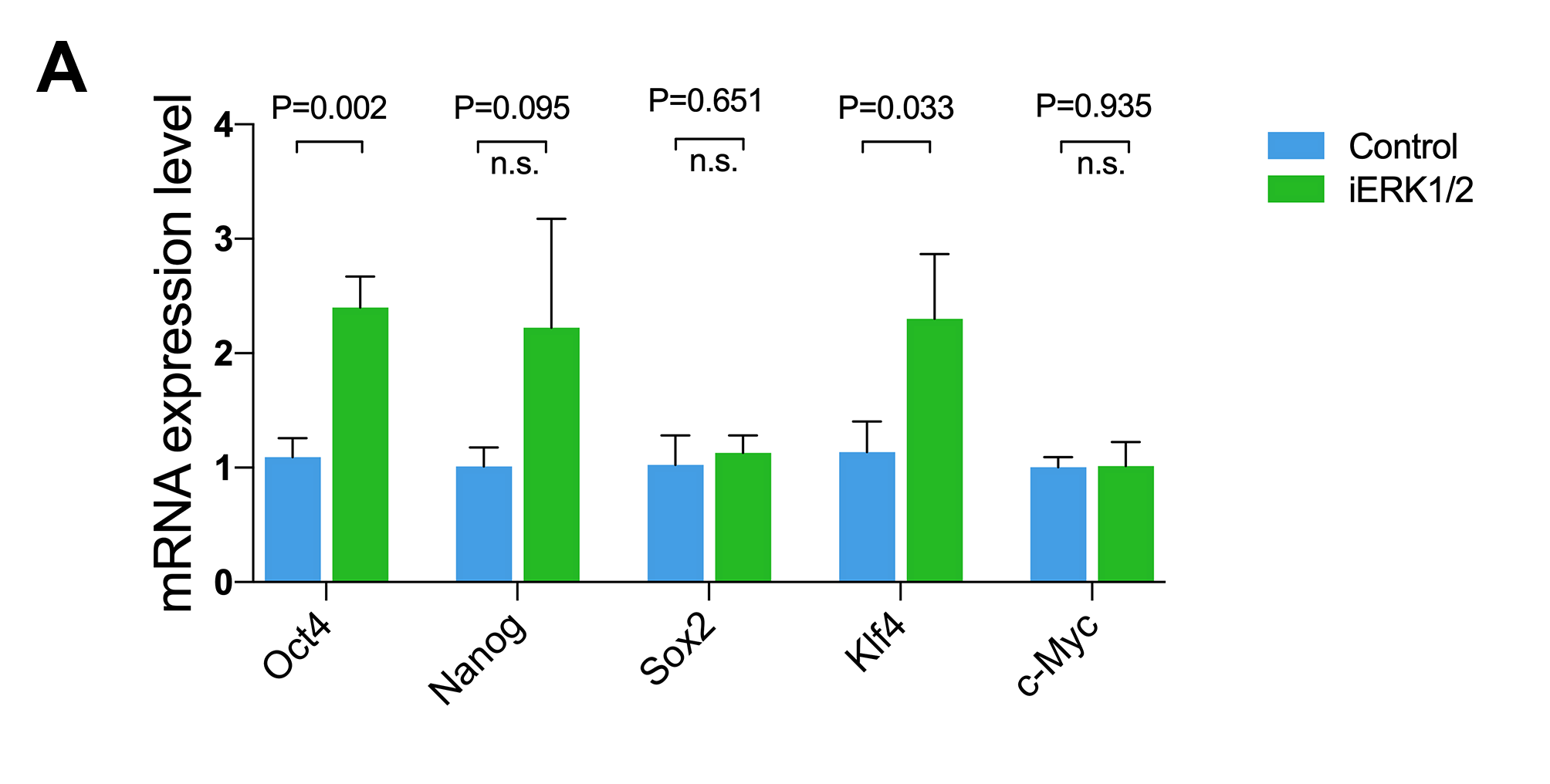
**

**Additional file1: Figure S3.** The expression of pluripotent genes in morula that zygotic ERK1/2 inhibited. **A** Statistical analysis was carried out using Student’s t-test (two-sided). Error bars indicate SD. P values are indicated.

**Additional file1 Figure S4**


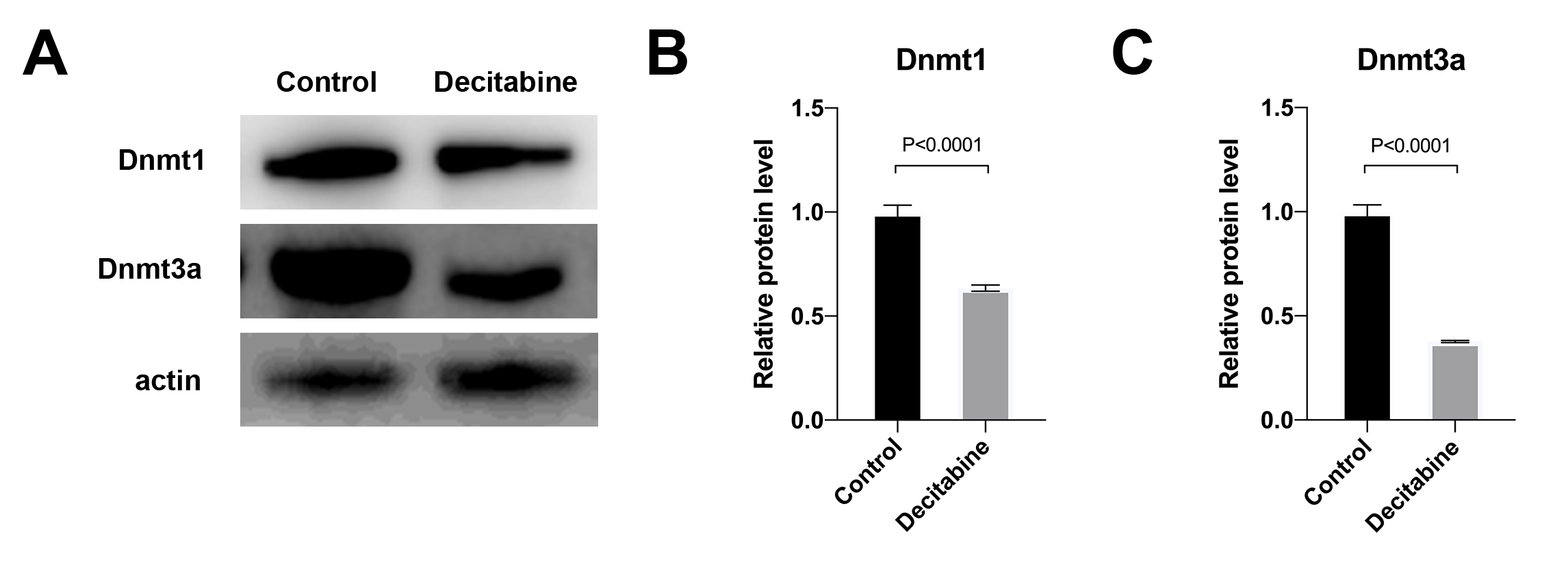


**Additional file1: Figure S4.** The expression of Dnmt1 and Dnmt3a in DNMT-inhibited zygotes. **A** Dnmt1 and Dnmt3a proteins expression in zygotes (8 hpf) following treatment with decitabine were verified by western blot analysis. Number of zygotes analysed for each group: control n=50; Decitabine-treated n=50. Statistical analysis of Dnmt1 (**B**) and Dnmt3a (**C**) in control and Decitabine-treated zygotes. Statistical analysis was carried out using Student’s t-test (two-sided). Error bars indicate SD. P values are indicated.
