# Supplementary material for: Regulation of paternal 5mC oxidation and H3K9me2 asymmetry by ERK1/2 in mouse zygotes
Source: Cell Biosci. 2022 Mar 7;12:25. doi: 10.1186/s13578-022-00758-x (PMC8900417; doi:10.1186/s13578-022-00758-x)
Supplement: Supplementary file 2 — Additional file 2: Table S1. Primers used for qRT-PCR analysis. [file 13578_2022_758_MOESM2_ESM.docx]

**Additional file2 Table S1.** Primers used for qRT-PCR analysis.

| Gene name | Forward primer | Reverse primer |
| --- | --- | --- |
| Oct4 | TTTGGAAAGGTGTTCAGCCAG | AAGGTTCTCATTGTTGTCGGCT |
| Nanog | ACCTGAGCTATAAGCAGGTTAAGAC | GTGCTGAGCCCTTCTGAATCAGAC |
| Sox2 | ACAGCTACGCGCACATGA | GTTCATGTAGGTCTGCGAGC |
| Klf4 | TGTGTCGGAGGAAGAGGAAGC | ACGACTCACCAAGCACCATCA |
| c-myc | CTGCGTGACCAGATCCCTGA | GCTTGTGCTCGTCTGCTTGAA |
| H19 | TGGAGTCCCGGAGATAGCTT | TCAGACGGAGATGGACGACA |
| Igf2 | CGTGGCATCGTGGAAGAGTG | GTACGGCCTGAGAGGTAGAC |
| GAPDH | CCAATGTGTCCGTCGTGGAT | TGCCTGCTTCACCACCTTCT |
